# Ruminal resistome of dairy cattle is individualized and the resistotypes are associated with milking traits

**DOI:** 10.1186/s42523-021-00081-9

**Published:** 2021-02-10

**Authors:** Ming-Yuan Xue, Yun-Yi Xie, Yi-Fan Zhong, Jian-Xin Liu, Le Luo Guan, Hui-Zeng Sun

**Affiliations:** 1 Institute of Dairy Science, Ministry of Education Key Laboratory of Molecular Animal Nutrition, College of Animal Sciences, Zhejiang University, Hangzhou, 310058 China; 2 Department of Agricultural, Food and Nutritional Science, University of Alberta, Edmonton, AB T6G 2P5 Canada

**Keywords:** Dairy cattle, Rumen, Metagenomics, Resistome, Microbiome

## Abstract

**Background:**

Antimicrobial resistance is one of the most urgent threat to global public health, as it can lead to high morbidity, mortality, and medical costs for humans and livestock animals. In ruminants, the rumen microbiome carries a large number of antimicrobial resistance genes (ARGs), which could disseminate to the environment through saliva, or through the flow of rumen microbial biomass to the hindgut and released through feces. The occurrence and distribution of ARGs in rumen microbes has been reported, revealing the effects of external stimuli (e.g., antimicrobial administrations and diet ingredients) on the antimicrobial resistance in the rumen. However, the host effect on the ruminal resistome and their interactions remain largely unknown. Here, we investigated the ruminal resistome and its relationship with host feed intake and milk protein yield using metagenomic sequencing.

**Results:**

The ruminal resistome conferred resistance to 26 classes of antimicrobials, with genes encoding resistance to tetracycline being the most predominant. The ARG-containing contigs were assigned to bacterial taxonomy, and the majority of highly abundant bacterial genera were resistant to at least one antimicrobial, while the abundances of ARG-containing bacterial genera showed distinct variations. Although the ruminal resistome is not co-varied with host feed intake, it could be potentially linked to milk protein yield in dairy cows. Results showed that host feed intake did not affect the alpha or beta diversity of the ruminal resistome or the abundances of ARGs, while the Shannon index (*R*^*2*^ = 0.63, *P* < 0.01) and richness (*R*^*2*^ = 0.67, *P* < 0.01) of the ruminal resistome were highly correlated with milk protein yield. A total of 128 significantly different ARGs (FDR < 0.05) were identified in the high- and low-milk protein yield dairy cows. We found four ruminal resistotypes that are driven by specific ARGs and associated with milk protein yield. Particularly, cows with low milk protein yield are classified into the same ruminal resistotype and featured by high-abundance ARGs, including *mfd* and *sav*1866.

**Conclusions:**

The current study uncovered the prevalence of ARGs in the rumen of a cohort of lactating dairy cows. The ruminal resistome is not co-varied with host feed intake, while it could be potentially linked to milk protein yield in dairy cows. Our results provide fundamental knowledge on the prevalence, mechanisms and impact factors of antimicrobial resistance in dairy cattle and are important for both the dairy industry and other food animal antimicrobial resistance control strategies.

**Supplementary Information:**

The online version contains supplementary material available at 10.1186/s42523-021-00081-9.

## Background

Antimicrobial resistance (AMR) is a major public health challenge and increases morbidity and mortality in humans and food-producing animals [1–3]. Both harmless and beneficial bacteria act as reservoirs of antimicrobial resistance genes (ARGs) [4, 5], and the ARGs can be transferred within the microbial community via the mechanism of horizontal gene transfer [6, 7]. Likewise, ARGs in livestock animals can be transmitted to humans through the food chain (e.g., dairy milk), and can enter the water and soil through runoff from manure [8, 9]. Therefore, investigating the ARGs in livestock animals is of great importance to address the issues of livestock industry sustainability and the public health concern of AMR. In ruminants, it has been widely reported that fecal shedding is a common route for AMR transmission in agriculture, with the microbes in feces representing reservoirs of ARGs [9–13] . Recent studies also indicated that the rumen microbiome carries a large number of ARGs as well, which may disseminate to the environment through saliva or the flow of rumen microbial biomass to the hindgut [14].

Previous studies have demonstrated that the microbes in the rumen and hindgut of cattle are reservoirs of ARGs [9–12], and the majority of studies focused on the impact of antimicrobial (therapeutic or subtherapeutic) administration. The effects of diet on the ARGs in the feces [13] and rumen [14] of antimicrobial-free cattle have been reported recently, and the results indicate that diet-driven dynamic changes of the microbiome could potentially modify the microbial resistome (the collection of all detected ARGs). However, the understanding of host effects (for instance, feed intake) on the microbial resistome in ruminants is still limited. Here, we speculated that the host effect of feed intake could be an important impact factor influencing the rumen resistome.

Our previous studies have revealed that the rumen microbiome is largely individualized and contributes to personalized milking traits of dairy cattle [15, 16]. Based on the fact that microbial ARGs are mainly structured by bacterial phylogeny [17, 18], it was speculated that ruminal ARGs could also be individualized in cattle with varied production. Inspired by the concept of enterotype in human studies (describing the distinct gut microbial composition types which are relevant in host phenotypes) [19], we speculated that animals also could be classified based on their ruminal resistome (defined as ruminal resistotype) and the resistotypes could be associated with cattle phenotypes.

To uncover the above knowledge gaps, we designed two studies that included a total of 49 lactating dairy cows, aiming to test the hypotheses that 1) the resistome is driven by host feed intake and 2) animals with different milking traits have distinctive ruminal ARG profiles. Rumen digesta samples from 33 and 16 lactating dairy cows were collected and employed in studies 1 and 2, respectively, to test the above hypotheses. Metagenomic sequencing of these samples was conducted to characterize the profiles of the ruminal resistome. We further explored the effects of feed intake on ARG profiles and associations between the ruminal resistome and milking traits. The current research provides a fundamental understanding of the ARGs in the rumen of dairy cattle and reveals the potential relationships between microbial ARGs and host production.

## Results and discussion

### Profiles and mechanisms of the ruminal resistome in lactating dairy cows

Shotgun metagenomic sequencing generated a total of 2,751,185,494 reads from rumen samples of 49 dairy cows (Supplementary Table S1). After quality control and removing host genes, 34,039,290 contigs were assembled. We first characterized the microbial profile, with 33,226,582 contigs annotated to RefSeq database. The rumen microbiome consisted of 93.38 ± 6.54% (mean ± standard deviation) bacteria, 4.58 ± 6.53% eukaryote, 1.35 ± 0.72% archaea, and 0.47 ± 0.34% viruses (Supplementary Fig. 1A). The dominant bacterial phyla included *Bacteroidetes* (53.98 ± 5.65%), *Firmicutes* (30.98 ± 6.15%), *Proteobacteria* (6.09 ± 4.65%), *Actinobacteria* (1.31 ± 0.84%), and *Spirochaetes* (0.52 ± 0.20%) (Supplementary Fig. 1B).

**Fig. 1:**
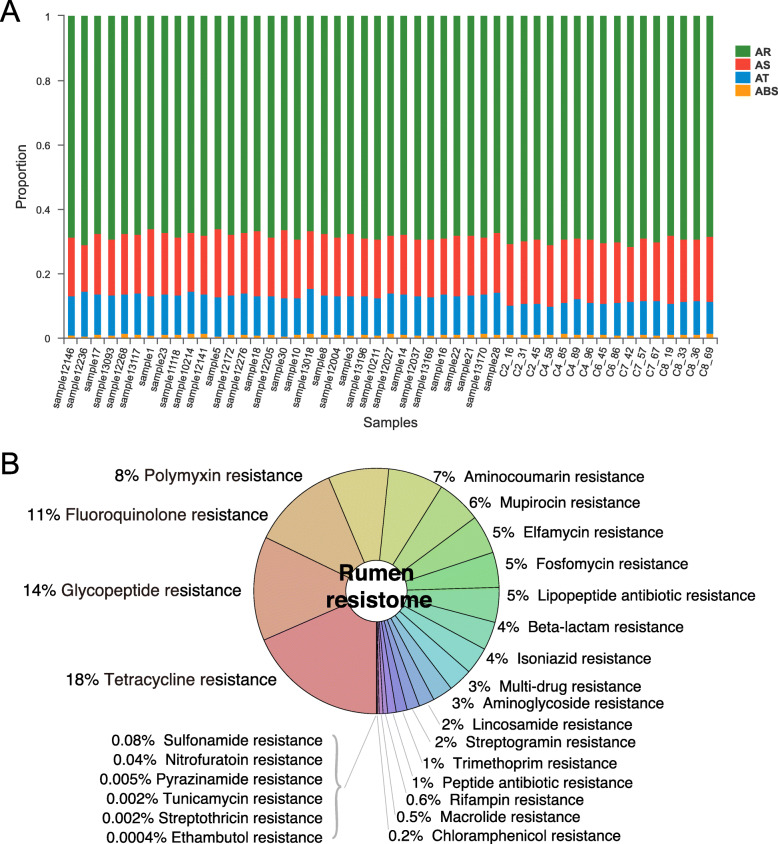
Composition of ruminal resistome in dairy cows. **a** Abundances of ARGs (antimicrobial resistance genes) per branch. ABS, Antimicrobial Biosynthesis; AT, Antimicrobial Target; AS, Antimicrobial Sensitive; AR, Antimicrobial Resistance. **b** Ruminal resistome composition summarized at the antimicrobial-class level

A total of 19,674,072 contigs were annotated to the Comprehensive Antimicrobial Resistance Database (CARD) database, and 343 ARGs were identified. The ARGs consisted of 68.6 ± 1.29% antimicrobial resistance genes (defined as AR in CARD), 18.8% ± 1.14% antimicrobial sensitive genes (AS), 11.5 ± 1.29% antimicrobial target genes (AT), and 1.13 ± 0.22% antimicrobial biosynthesis genes (ABS) were identified using CARD (Fig. 1a). The ruminal resistome of lactating dairy cows in this study was predicted to confer resistance to 26 different classes of antimicrobials (Fig. 1b). Previous studies of bovine microbial resistome mainly focused on fecal microbiota and found that feces serve as the primary route of AMR contamination from cattle to the environment [9, 13]. Our results, together with a previous study [14], indicate that the rumen microbiome also carries a large number of genes that confer resistance to different antimicrobial classes in cattle, and these genes may disseminate to the environment through animal saliva or to the feces through the gastrointestinal tract [20]. We found that genes conferring resistance to tetracycline in the rumen were the most abundant (18%) (Fig. 1b), and this result is consistent with previous studies [14, 21]. It has been demonstrated that genes carrying tetracycline resistance are present after cattle are born and increase during nursing, although the animals have been antibiotic-free since birth [13]. The high prevalence of such resistance genes in antimicrobial-free animals may be due to the presence of commensal bacteria naturally carrying ARGs and being part of core bacteria in antimicrobial-free animals after the long-term use of subtherapeutic doses of tetracycline in dairy veterinary practice [22]. Additionally, a high background of AMR in the environment, such as basin water, manured agricultural soil and urban sewage [9], could also influence the ruminant overall resistome.

**Fig. 2:**
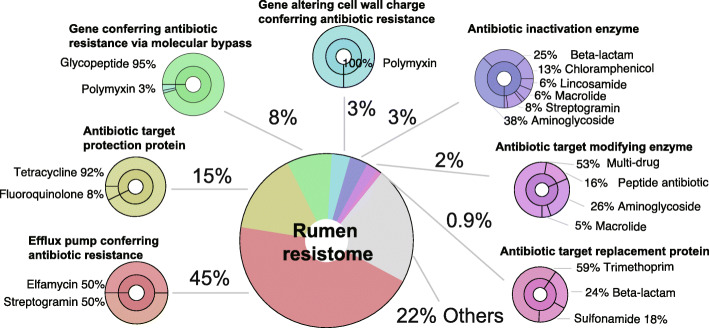
Antimicrobial resistance mechanisms in the rumen microbiome. The rumen microbiome in dairy cows exhibited broad antimicrobial resistance mechanisms classified in 7 categories. The classes of antimicrobials observed in the category of each mechanism are presented

The mechanisms of rumen antimicrobial resistance to different classes of antimicrobials were then identified (Fig. 2). In our ruminal resistome dataset, approximately 45% of the ARGs were linked to the mechanism of antimicrobial efflux, conferring resistance to elfamycin and streptogramin. Among these sub-terms conferring resistance by antimicrobial efflux, 40% were subunit of efflux pump conferring antibiotic resistance, 38.26% were resistance-nodulation-cell division (RND) antibiotic efflux pump, 10.43% were ATP-binding cassette (ABC) antibiotic efflux pump, 7.83% were major facilitator superfamily (MFS) antibiotic efflux pump, and 3.48% were multidrug and toxic compound extrusion (MATE) transporter (Supplementary Table S2). Fifteen percent of the ARGs conferred resistance by encoding antimicrobial target protection proteins, including mostly tetracycline resistance genes. Eight percent of the ARGs were involved in the mechanism of molecular bypass, including most glycopeptide antimicrobial resistance genes. Approximately 3% of the ARGs alter cell wall charge to confer resistance, and all these genes are associated with polymyxin resistance. Another 3% of the ARGs encoded antibiotic inactivation enzymes (Fig. 2). In addition, 22% of ARGs were considered to act through other mechanisms, since no mechanistic information of these ARGs was identified based on CARD.

The mechanism profiles of antimicrobial resistance identified in the current study revealed that antimicrobial efflux was the major route for the transmission of AMR in rumen microbiome. However, some mechanisms of AMR are very complex, making it difficult to obtain such information from the current antimicrobial resistance databases. For instance, some resistance occurs from epistatic relationships between multiple genes [23], many resistances can arise via overexpression of structural genes such as genes encoding efflux pumps, and some ARGs may not always be expressed [24]. Therefore, it is still challenging to accurately reveal the mechanisms of the ruminal resistome by predicting phenotypic antimicrobial resistance from genotypic antimicrobial resistance genes [7]. Identifying and verifying such resistance mechanisms may require further mRNA-level and protein-level measurements.

### Assigning the ARG-containing contigs to bacterial taxonomy

**Fig. 3:**
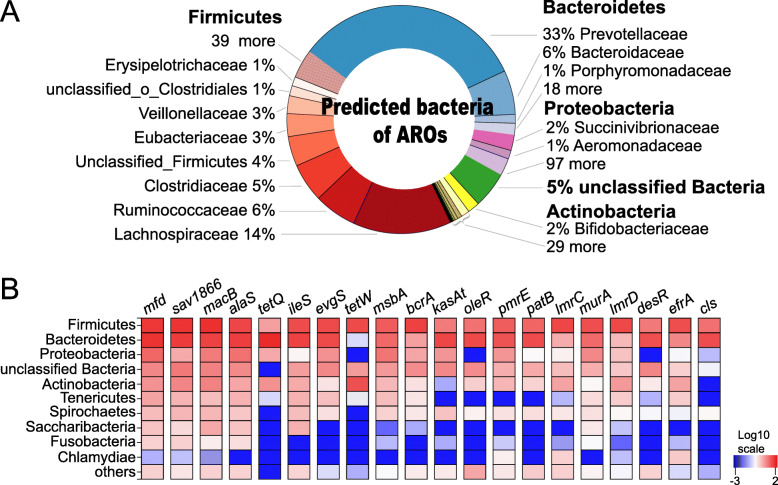
The predicted bacterial taxa of the ruminal resistome and the relative abundances of related resistance genes. **a** Composition of observed bacterial taxa predicted by the ruminal resistome summarized at the phylum and family levels. **b** Distributions of ARGs in the phyla of predicted rumen bacteria. The top 10 phyla are displayed, with the remaining bacterial phyla included in the “others” category. The distributions of the ARGs are presented as coloured boxes, with the top 20 resistance genes listed. ARGs: antimicrobial resistance genes

The ARG-containing contigs of these 49 samples were assigned to bacterial taxonomy to predict the bacterial origin of the observed ARGs, with 19,204,342 ARG-containing contigs assigned to bacteria (97.61% of contigs harbouring ARGs, Supplementary Table S1). The ARG-containing bacteria belonged to 7 phyla, accounting for 99% of the total ARG abundances (Fig. 3a). Taxa of the phylum *Firmicutes* (relative abundance of 42.4 ± 7.71%, mean ± standard deviation) showed the largest proportion of bacteria harbouring ARGs, with most of the ARGs detected in the families *Lachnospiraceae* (13.9 ± 2.34%), *Ruminococcaceae* (6.13 ± 2.21%), and *Clostridiaceae* (5.53 ± 1.12%). The ARG-containing taxa of the phylum *Bacteroidetes* (41.9 ± 7.03%) mainly belonged to the families *Prevotellaceae* (32.6 ± 6.82%) and *Bacteroidaceae* (6.39 ± 1.33%). The ARG-containing taxa of the phylum *Proteobacteria* (5.82 ± 5.01%) mainly belonged to the families *Succinivibrionaceae* (2.17 ± 1.92%), *Aeromonadaceae* (1.23 ± 1.14%), and *Enterobacteriaceae* (0.77 ± 0.95%) (Fig. 3a). At the genus level, the predominant ARG-containing bacterial genera (relative abundance > 1% and existed in > 50% of all the samples) accounted for 74.7% of all ARG abundances, with *Prevotella* (30.6 ± 6.41%), *Bacteroides* (6.37 ± 13.36%), unclassified *Lachnospiraceae* (5.50 ± 1.23%), *Clostridium* (5.20 ± 1.18%), and unclassified bacteria (5.23 ± 0.95%) being the most abundant. Large inter-animal variations in the abundances of ARG-predicted genera were observed, with the coefficient of variances (CVs) ranging from 20.3 to 210% (Table 1), indicating that the ARGs in the rumen microbiome are individualized.

**Table 1:** Relative abundances of predominant ARG-predicted bacterial genera

Genera	Mean	Standard deviation	Coefficient of variances
*Prevotella*	30.57%	6.41%	21.62%
*Bacteroides*	6.37%	1.36%	210.06%
*unclassified (f) Lachnospiraceae*	5.50%	1.23%	24.55%
*Clostridium*	5.20%	1.18%	23.63%
unclassified *(d) Bacteria*	5.23%	0.95%	20.31%
unclassified *(p) Firmicutes*	4.39%	1.34%	28.67%
*Ruminococcus*	3.89%	1.58%	37.43%
*Butyrivibrio*	3.41%	0.63%	34.00%
*Eubacterium*	3.16%	0.97%	24.75%
*Bifidobacterium*	1.63%	0.74%	67.31%
*Selenomonas*	1.56%	0.50%	43.23%
unclassified *(o) Clostridiales*	1.23%	0.40%	39.14%
*Roseburia*	1.11%	0.31%	33.87%
*Paraprevotella*	1.01%	0.31%	32.66%
*Phascolarctobacterium*	0.91%	0.36%	35.85%
*Alistipes*	0.99%	0.32%	34.40%
unclassified (f) *Ruminococcaceae*	0.91%	0.26%	32.14%
*Blautia*	0.87%	0.23%	28.19%
*Aeromonas*	0.91%	1.04%	81.33%
*Ruminobacter*	0.87%	0.74%	100.63%
*Faecalibacterium*	0.75%	0.29%	69.74%
*Lactobacillus*	0.68%	0.17%	34.77%
*Oribacterium*	0.62%	0.22%	31.28%
*Succinatimonas*	0.62%	0.62%	73.60%
*Olsenella*	0.47%	0.43%	106.08%
*Mycoplasma*	0.52%	0.20%	70.25%
*Campylobacter*	0.54%	0.20%	37.48%
*Pseudobutyrivibrio*	0.47%	0.17%	38.91%
*Treponema*	0.45%	0.27%	52.51%
*Tolumonas*	0.42%	0.37%	78.35%
unclassified *(o) Bacteroidales*	0.32%	0.24%	91.34%
*Kandleria*	0.27%	0.43%	134.62%
*Dialister*	0.27%	0.36%	162.23%
*Sharpea*	0.26%	0.28%	125.98%
*Desulfosporosinus*	0.23%	0.19%	103.78%
others	13.39%	1.87%	50.35%

Several of the predominant bacterial genera predicted to carry ARGs, including *Prevotella*, *Clostridium* and *Ruminococcus* [21], have been considered the core bacteria in the rumen of dairy cows and other ruminant species [25–28]. *Prevotella*, which utilizes starch and proteins to produce succinate and acetate, is commonly considered the most predominant bacterial genus in the rumen of adult dairy cows [29]. Our previous study reported that *Prevotella* positively contributed to volatile fatty acid production in the rumen and milk protein yield [16], and other studies have also reported that the OTUs belonging to *Prevotella* were associated with the feed efficiency [30] and milk protein yield [31] of dairy cows. *Ruminococcus* plays roles in breaking down fibrous plants and producing acetate and has been identified as the second predominant bacterium in dairy cows fed a corn-based high-grain diet [27]. *Clostridium* is a cellulolytic, proteolytic and amylolytic bacterium [32]; *Clostridium* has been reported to be more abundant in the rumen of cows with lower levels of milk protein yield [16] and negatively correlated with milk protein content [31]. The identification of these core bacteria as predominant ruminal ARG reservoirs suggests the high presence of ARGs in the rumen ecological niches of dairy cows. These predominant microbial ARG reservoirs identified in the rumen are bacterial members that play vital roles in feed fermentation and volatile fatty acid production, which subsequently determine or largely affect host phenotypes. This suggests that when we consider improvement strategies for animal production performance that involve manipulating the rumen microbiome, the presence of ruminal ARGs should be taken into consideration.

When the ARG distributions in different bacterial phyla were compared based on the distributions of the top 20 most abundant ARGs in the top 10 most abundant phyla, the most dominant ARGs (counts per million reads [CPM] > 500 in over 60% of the samples), including *mfd*, *sav*1866, *macB*, and *alaS*, were distributed in the majority of the abundant bacterial phyla (Fig. 3b). *mfd* encodes the transcription-repair-coupling factor and confers resistance to fluoroquinolone antimicrobials [33]. The *sav*1866 and *macB* genes confer resistance through the mechanism of the efflux pump, with *sav*1866 encoding a multidrug export ATP-binding/permease protein [34] and *macB* encoding a macrolide export ATP-binding/permease protein, which is part of the efflux system MacAB-TolC pump [35]. The *alaS* gene encodes the alanine-tRNA ligase [36] and confers resistance to aminocoumarin. The high abundances of these ARGs in the current study indicate the prevalence of these ARGs in the ruminal microbiome of lactating dairy cows without recent antimicrobial usage (animals used in this study received no therapeutic or prophylactic antimicrobial treatment since the first day of lactation).

Although most of the highly abundant bacterial phyla were predicted to confer resistance to at least one antimicrobial, the resistance between different bacterial phyla showed high variation, and a higher prevalence of ARGs was identified in the predominant phyla (Fig. 3b). For example, 20 and 19 out of the top 20 most abundant ARGs were identified in bacterial taxa belonging to *Firmicutes* and *Bacteroidetes*, while less than 5 out of the top 20 most abundant ARGs were identified in *Fusobacteria* and *Chlamydiae*. A previous study that analysed rumen bacterial and archaeal genomes also revealed that ARGs were more prevalent among members of the phyla *Firmicutes*, *Proteobacteria*, *Bacteroidetes*, and *Actinobacteria* in different ruminant species [21]. In ovine, ruminal ARGs were identified mostly within the phyla *Firmicutes* and *Proteobacteria* [37]. These previous works together with the current study indicate that although ARGs are widely distributed across rumen bacterial taxa, resistance to specific antimicrobials is more prevalent in the above bacterial phyla.

### Effect of host feed intake on ruminal resistome

We then characterized whether the ruminal bacteriome and resistome are affected by the host physiological effect of feed intake. Cows with the highest dry matter intake (DMI, *n* = 10) and lowest DMI (*n* = 10) in study 1 were selected to detect whether the host feed intake could affect the ruminal resistome. Power calculation revealed that the sample size of 10 enabled 99% power and a type I error of 5%, based on a t-test of the DMI. The bacteriome was compared between high and low DMI groups based on Beta diversity and relative abundances, and barely difference was found between the two DMI groups (Supplementary Fig. 2).

**Fig. 4:**
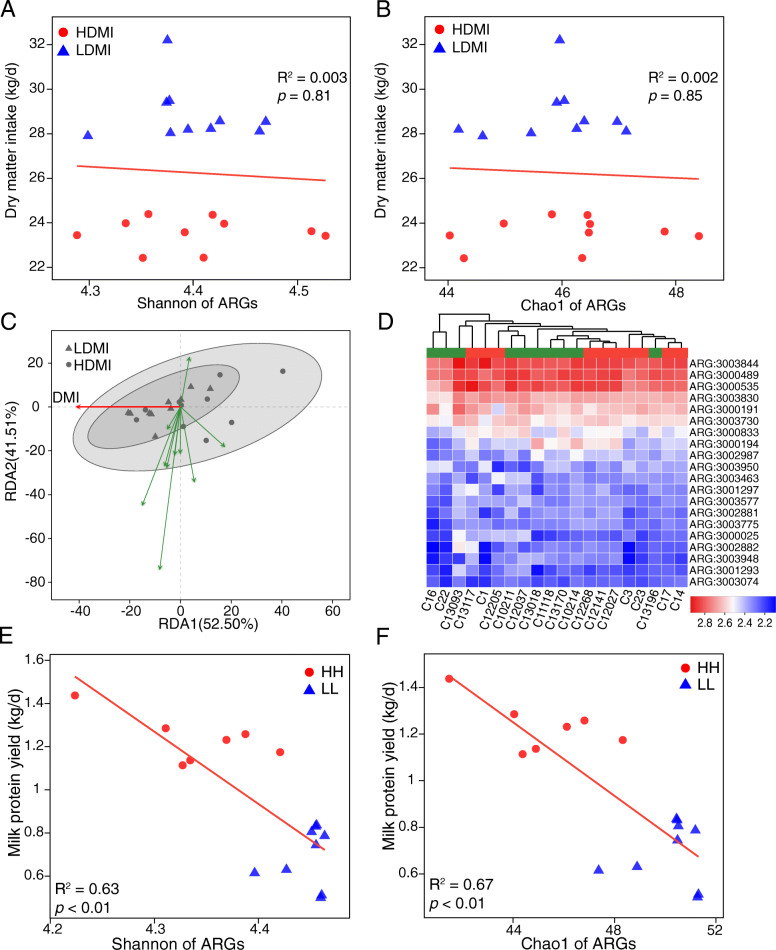
Ruminal resistome profiles of cows with different feed intake and cows with different milking traits. Spearman’s rank correlations between Shannon index (**a**) and Chao 1 richness (**b**) of ARGs and dry matter intake. **c** Biplot of the redundancy analysis showed relationships between ARGs and dry matter intake. The top 10 most abundant ARGs were used in this analysis. HDMI, cows with the highest dry matter intake (*n* = 10); LDMI, cows with the lowest dry matter intake (*n* = 10). **d** Heatmap of the abundances of the top 20 ARGs in each sample. The abundances (CPM, counts per million) of ARGs were log10- transformed. The cows were clustered and coloured by different groups (green, high intake; red, low intake). The animals used in the above analysis were all selected from study 1. Spearman’s rank correlations between Shannon index (**e**) and Chao 1 richness (**f**) of ARGs and milk protein yield. The animals used were selected from study 2. *R*^*2*^ = correlation coefficient. ARGs: antimicrobial resistance genes

The correlation analysis showed no significant correlations between the alpha diversity (Shannon and Chao1 indices) of ARGs and DMI (*P* > 0.80, Fig. 4a and b). The redundancy analysis (RDA) plot illustrated that the ruminal resistome in cows with different DMIs was not separable, and the relationship between the abundant rumen ARGs and host feed intake was close to zero (Fig. 4c). The heatmap and the clustering based on the abundances of the 20 most dominant ARGs revealed no clusters between different feed intake groups (Fig. 4d). The abundance of each ARG was also compared between the two DMI groups, while no differential ARG was observed (permutational multivariate ANOVA [PERMANOVA], *P* = 0.90) (Supplementary Table S3).

Many factors can affect the rumen resistome, for example, previous microbial ARG studies in cattle using culture-based methods mainly focused on the influence of antimicrobial administration (therapeutic or subtherapeutic administration) on microbial ARGs in feces [38]. Recent studies using sequencing-based methods reported the effect of diet on the microbial resistome in feces [13] and rumen [14] of antimicrobial-free cattle, suggesting that the diet-driven dynamic changes of the microbiome could potentially modify the gastrointestinal resistome. Here, we considered one of the important host physiological effects, feed intake, on rumen microbial ARGs. It is known that feed intake affects microbial growth due to the differential amounts of available substrates for the growth of microbiota [39] and the varied rumen passage rate [40–42]. However, our results showed that both the ruminal bacteriome and resistome did not co-vary with host feed intake. The primary effect on the microbial composition and ARGs was diet ingredients [13, 14] rather than the effect of feed intake or breed [14], suggesting that the external stimulus may be a major cause of changes in the gastrointestinal resistome. Although the host effect of feed intake may not be the major cause of resistome changes, other host effects, such as genetics, which has been confirmed to regulate the rumen microbiome [43–45], should be considered and linked to the microbial resistome in future studies.

### The relationship between the ruminal resistome and milking traits

We then investigated whether the ruminal resistome differs between cows with different milking traits, which were fed the same corn-based high-grain diet and under the same management condition. The ruminal resistome of 16 lactating dairy cows in study 2 was separated into two significantly separable clusters based on different milk protein contents (PERMANOVA *P* < 0.01, Supplementary Fig. 3A) and milk protein yield (PERMANOVA *P* < 0.01, Supplementary Fig. 3B), while no clear cluster based on milk fat contents (PERMANOVA *P* = 0.23) or lactose contents (PERMANOVA *P* = 0.35) was observed (Supplementary Fig. 3C and D). Significantly positive correlations between Shannon index (*R*^*2*^ = 0.63, *P* < 0.01) and richness (Chao1 index, *R*^*2*^ = 0.67, *P* < 0.01) of the ruminal resistome and milk protein yield were observed (Fig. 4e and f).

Based on the above results, the 16-cow dataset was divided into two groups according to milk protein yield, including 7 cows with high milk protein yield (HH, milk protein yield > 1.11 kg/d) and 9 cows with low milk protein yield (LL, milk protein yield < 0.84 kg/d). Power calculations revealed that the sample size enabled 88% power and a type I error of 5%, based on a t-test of milk protein yield. The comparison of rumen bacteriome between HH and LL groups showed clear clustering based on Beta diversity, together with significant differences in abundant bacterial species (Supplementary Fig.4).

**Fig. 5:**
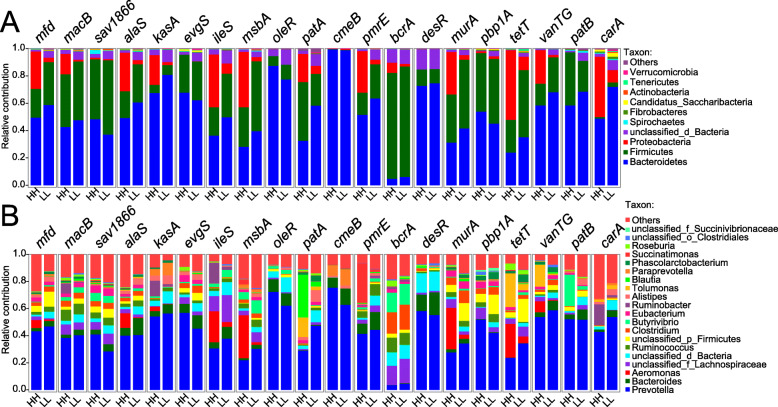
Distributions of ARGs annotated to bacterial taxa in the rumen of cows with different milk protein yield. The proportion of ARG contigs annotated to the top 10 most abundant bacterial phyla (**a**) and top 20 most abundant bacterial genera (**b**) are shown in the bar plots. ARGs: antimicrobial resistance genes

**Fig. 6:**
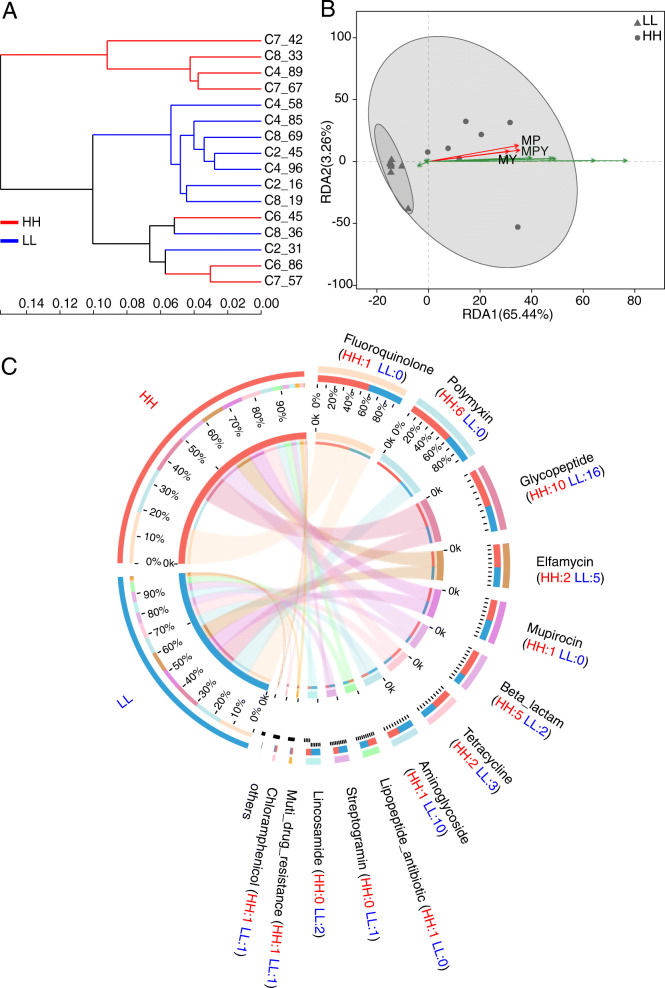
Distinguishable ruminal resistome between high- and low-milk protein yield dairy cows. **a** The clustering of ARGs obtained from dairy cows with high and low milk protein yield based on Bray-Curtis dissimilarity. **b** Biplot of the redundancy analysis showed relationships between ARGs and milk protein content (MP), milk yield (MY), and milk protein yield (MPY). The top 10 most abundant ARGs were used in this analysis and are indicated by green arrows. **c** Significantly different ARGs categorized by antimicrobial classes. Each antimicrobial class was represented by an individual colour in the external circle of the plot. The numbers of significantly higher ARGs in each group belonging to each antimicrobial class are shown in the plot. ARGs: antimicrobial resistance genes

The distributions of the predominant predicted ARG-carrying bacterial phyla and genera in the ruminal resistome of each group (top 20 ARGs represented) showed distinguishable patterns between the HH and LL groups (Fig. 5 and Supplementary Table S4). The Bray-Curtis dissimilarity-based clustering analysis showed a trend of separation between the cows with different milk protein yields (Fig. 6a), with the RDA revealing that the 7 most abundant ARGs (indicated by the green arrows) positively contributed to the separation and were positively correlated with the milk protein content and yield (indicated by the red arrows, Fig. 6b). The abundances of ARGs in the HH and LL groups were then compared; 128 ARGs (accounting for 44.76% of the total ARG number) had significantly different abundances (FDR < 0.05, PERMANOVA *P* < 0.01). These significantly different ARGs conferred resistance to 13 classes of antimicrobials (Fig. 6c). The most abundant ARG, *mfd*, conferring resistance to fluoroquinolone, was significantly higher in the HH group (FDR = 0.008) (Supplementary Table S5). The rumen microbiome of the HH cows also harboured higher abundances of genes conferring resistance to polymyxin, including *pmrE*, *pmrF*, *pmrC*, and *pmrA* (FDR < 0.05). The rumen microbiome of the LL cows had more enriched genes conferring resistance to glycopeptide antimicrobials, elfamycin, and aminoglycoside antimicrobials (Fig. 6c). Notably, among the top 20 differential ARGs, 17 had significantly higher abundances in the rumen of the HH cows, while only 3 low-abundance ARGs were enriched in the rumen of the LL cows (Supplementary Table S5). Different ruminal ARG patterns between the cows with different milk protein yields suggest that the improvements in production could have a negative impact on the ruminal AMR, although no antimicrobial treatment was used for promoting production.

**Fig. 7:**
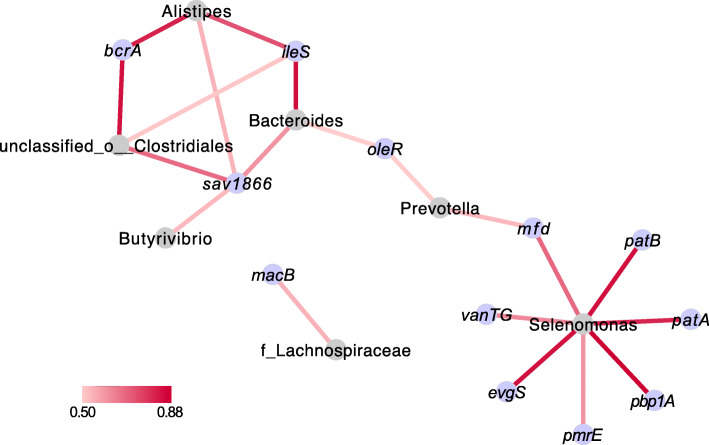
Co-occurrence network of abundant bacterial genera and ARGs. Abundant bacterial genera (top 10) and ARGs (top 20) of animals in study 2 were selected and used in the co-occurrence analysis. Only strong positive relationships (coefficient > 0.5 and *P* < 0.05) were displayed in the network

Our current study together with previous studies [15, 16, 46] showed that both the rumen bacteria compositional patterns and functional patterns (Supplementary Fig. 5) were different between the HH and LL cows, and several dominant bacteria contributed to host milk protein yield. The results in the current study showing different microbial ARG patterns make us speculate that these dominant bacteria may co-occur with specific ARGs. For example, our previous study reported that the abundance of *Prevotella* was significantly higher in the rumen of HH cows and that *Prevotella* positively contributed to volatile fatty acids and milk protein yield [16]. This taxon was also an important ARG reservoir which include the most predominant ARG, *mfd*, in the current study. The higher prevalence of *Prevotella* and *mfd* in the rumen of HH cows suggests the potential co-occurrence between this bacterial taxon and ARG, which was confirmed by the co-occurrence analysis of bacterial genera and ARGs (Fig. 7). The co-occurrence analysis also revealed positive relationships between *Selenomonas* and several ARGs, including *mfd*, *patB*, *patA*, *pbp1A*, *pmrE*, *evgS*, and *vanTG* (Fig. 7). The identification of above potential co-occurrences will provide evidence for balancing resistome prevention and microbiome manipulation for lower AMR and better production in dairy cows.

### The ruminal resistotypes were associated with milk protein yield

**Fig. 8:**
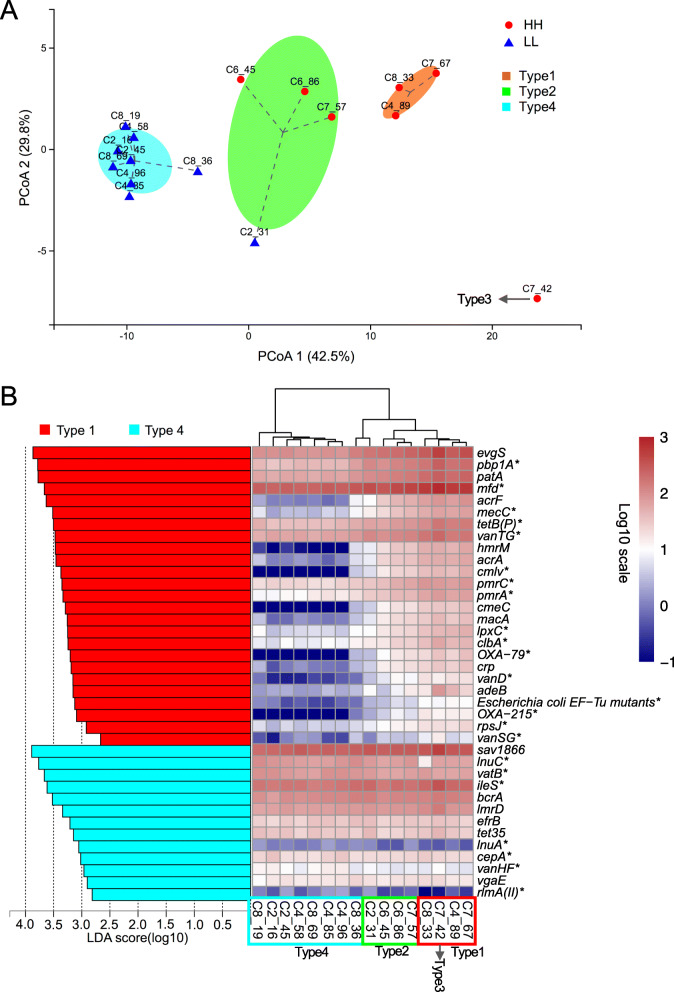
Stratification of the ruminal resistome composition in study 2. **a** The principal coordinate analysis of the ruminal resistome showed four resistance types (resistotypes) among the 16 dairy cows. **b** The significantly different ARGs among the four resistotypes tested by LDA effective size analysis, with LDA > 2 being considered significantly different. Differential ARGs among the four ruminal resistotypes were only found to be enriched in Type1 and Type4. The heatmap shows the abundances (log10-transformed reads per million) of each differential ARG. * Represents the ARGs that were also found to be significantly different between high- and low-milk protein yield groups. ARGs: antimicrobial resistance genes

Inspired by the concept of enterotype [47] and human intestinal resistotypes [48], we investigated the classification of animals based on their ruminal resistome and defined such classification as the ruminal resistotype. In our study, the 16 dairy cows were classified into 4 subpopulations based on the ruminal resistome (Fig. 8a), which was defined as the ruminal resistotype in the current study. Interestingly, nearly all the LL resistomes (8 out of 9) were classified into Type4 and were distinctly separated from the other three resistotypes, with the predominant ARGs in Type4 animals being *mfd* (5.84%) and *sav*1866 (5.45%). The LL cows were more likely to be distinguished from a cohort of animals, potentially due to their distinctive pattern of predominant ARGs (Supplementary Table S6). The other three resistotypes (Type1, 3 animals; Type2, 4 animals; and Type3, 1 animal) were observed in all HH cows and one LL cow (Fig. 8a).

To detect the key ARG drivers for different resistotypes, we performed LEfSe analysis on the ARGs based on the classification of four resistotypes. Significantly different ARGs among the four resistotypes were observed (Fig. 8b). The key ARG drivers were only found to be significantly enriched (LDA > 2) in Type1 (25 ARGs) and Type4 (13 ARGs), and such drivers were not found to be enriched in Type2 and Type3 (Fig. 8b). The heatmap revealed differential patterns of these ARG abundances and separations among the 4 resistotypes (Fig. 8b). Notably, 23 of the 38 ARG drivers were also found to be significantly different in cows with different milk protein yield (Fig. 8b).

The most predominant ARGs (including four ARGs with CPM > 100) were considered the most contributing drivers to the different resistotypes. The most abundant gene, *mfd* (mutation frequency decline), encodes a transcription-repair coupling factor involved in strand-specific DNA repair, conferring resistance to fluoroquinolone antimicrobials [33]. In addition to the most abundant *mfd*, other predominant ARGs, including *pbp1A*, *vanTG*, and *iles*, were also considered the most important critical drivers for different resistotypes. It has been demonstrated that PBP1A (penicillin-binding protein in *Streptococcus pneumoniae* isolates) possesses multiple substitutions that are highly associated with a reduced affinity for penicillin [49]. The *vanTG* gene (a *vanT* variant) confers resistance to vancomycin and resulted from the replacement of the target of peptidoglycan precursors [50]. The *Bifidobacteria* intrinsic *iles* gene (encoding isoleucyl-tRNA synthetase) confers resistance to mupirocin due to the key amino acid residues of the *iles* protein and is crucial for conferring a mupirocin resistance phenotype to *Bifidobacteria* [51]. We assumed that the above ARG-driven variations in animal production in the current study could be attributed to the variations in the microbial community, as microbial AMR is mainly structured by the bacterial community [18]. This possibility is supported by the current study (Fig. 5) and our previous studies, showing that the bacterial richness and the relative abundances of several bacterial taxa significantly differed between cows with different milk protein yield [15, 16]. The ARG drivers revealed in the current study suggest that these key ARGs may potentially co-vary with the microbial biomarkers identified in the rumen of cows with different milk quality, and further study is needed to confirm their relationships and underlying mechanism.

In the current study, the dairy cows were classified into different resistotypes which were associated with individualized phenotype of milk protein yield (study 2) rather than DMI (study 1, Supplementary Fig. 6). A human study also reported the clustering of subjects from a cohort of 663 people into 6 resistotypes, and these resistotypes were connected to enterotypes [48]. However, the causal effects behind such classification still need to be further explored. Due to the fact that different parity in our study could affect lactation performance and also chances to receive therapeutic treatment, the impact factor of parity should be considered in future study validating the association between resistotypes and milking traits. Also, although the power calculation revealed that our sample size in study 2 enables 87.5% power, the sample size of 16 is still relatively limited, indicating that future study should include a larger number of animals. Moreover, many factors affect the rumen microbiome, including diet [52], genetics [43], age [53], and lactation stage [15], indicating that the ruminal resistome could also be affected by these factors. The ruminal resistome composition and the resistotypes identified in the current study provide information about the ruminal resistome of mid-lactating Holstein dairy cows that were fed a corn-based high-grain diet. Future studies that examine the effects of other impact factors on ruminal resistome are required to identify whether ruminal resistotypes differ between breed types, lactation stage, or diet.

Majority of previous studies investigating the ARGs in rumen and hindgut have focused on the impact of antimicrobial (therapeutic or subtherapeutic) administration rather than focusing on the ARGs in antimicrobial-free animals. In our study, although the animals were free of antimicrobials during the experimental period and received no therapeutic or prophylactic antimicrobial treatment since the first day of lactation, this does not mean that animals in our study were not exposed to antimicrobials since birth. Additionally, the detection of ARGs at the DNA level detected in our study may not accurately reveal AMR phenotypes, since DNA can be released from dead microbes, and resistance can occur via overexpression of normal genes [7]. These suggest that transcriptional measurements focusing on absolutely antimicrobial-free animals are required for further comprehensive detection and validation.

## Conclusions

The current study provides insights into ARGs in the rumen of lactating dairy cows. Although no antimicrobials were given to the cattle during the experimental period in our study, the ruminal resistome conferred resistance to 26 antimicrobial classes, suggesting that the rumen microbiome serves as a reservoir for a high richness of AMR. Moreover, the analysis of the host effect on the ruminal resistome reveals that the resistome is not driven by the varied feed intake of the host, while the ruminal resistome compositions were different in cows with different milk protein yield. The resistomes could be classified into several resistotypes, which were driven by specific ARGs and associated with milk protein yield in dairy cows. These results indicate that the changes in the rumen microbiome composition could not only affect milking protein yield but also affect the ruminal resistome. In sum, our study uncovered the prevalence of ruminal ARGs in the rumen and the host-ruminal resistome interactions of dairy cows, providing fundamental knowledge and evidence for interventions to reduce AMR and regulate ARGs in ruminant livestock.

## Methods

### Animals, samples, and DNA extraction

The experimental protocol was approved by the Animal Care Committee of Zhejiang University (Hangzhou, China). All the animals used in two studies were free of antimicrobials during the experimental period and received no therapeutic or prophylactic antimicrobial treatment since the first day of lactation. The animals within each study were fed the same diet and under the same management condition. These two studies were previously designed and revealed that the host effect of feed intake is a crucial impact factor influencing rumen microbial metabolism (protein synthesis) and subsequently milk protein synthesis (study 1) [54], with individualized rumen microbiome playing important roles in regulating milk protein yield of dairy cow (study 2) [16, 46]. Power calculations revealed that sample sizes in these two studies enable 87.5% power and a type 1 error of 5%, based on t test of phenotypic parameters [46, 54].

In study 1, a cohort of mid-lactating Holstein dairy cows (parity = 2.48 ± 0.62, mean ± standard deviation) raised on a commercial dairy farm (Hangzhou, China) were selected for DMI measurements to detect the impact of feed intake on ruminal ARGs. This cohort included only healthy animals which were free of antimicrobials during the experimental period and received no therapeutic or prophylactic antimicrobial treatment since the first day of lactation, with 6 unhealthy animals treated with antimicrobials during the experimental period excluded. A total of 33 animals were finally left for further measurement. The cows were fed a total mixed ration that was formulated to produce 35 kg of milk per day with 3.25% milk protein as reported previously [54]. The milking traits were not different in animals with different DMIs [54]. The feed intake data were recorded using automatic weighting troughs (Roughage Intake Control System, Marknesse, Netherland), and the DMI was calculated as described previously [54].

In study 2, another 16 mid-lactating Holstein dairy cows (parity = 2.94 ± 1.34, mean ± standard deviation) were selected from a cohort of 374 healthy mid-lactating Holstein dairy cows raised on another commercial dairy farm (Hangzhou, China) based on our previous milking trait measurements to detect whether the resistome is individualized in cows with different milking traits [46]. In this study, 7 cows with the highest-milk protein yield (HH, cows with both the highest milk yield and protein content) and 9 cows with the lowest-milk protein yield (LL, cows with both the lowest milk yield and protein content) were selected and used for further analysis. Milk yield were > 34.5 kg/d for the HH and < 31 kg/d for the LL cows, and the milk protein content were > 3.20% for the HH and < 2.90% for the LL cows, respectively. These cows had different milking traits, including milk yield, milk protein content, and milk protein yield (milk yield × milk protein content) [16]. Cows were fed the same diet with a concentrate-to-forage ratio of 57:43 (dry matter basis) as described previously [55]. Rumen microbial profile of the 16 dairy cows has been reported previously, showing that several *Prevotella* species were significantly more abundant in the rumen of cows with higher milk protein yield, while methanogen were significantly lower [46].

Rumen digesta contents were sampled using oral stomach tubes at the same time of the sampling day of each study [56]. To reduce saliva contamination, we inserted the oral stomach tubes into the central rumen and discarded the first 150 mL of rumen fluid during sampling, and shortened the sampling time as well [56]. Total genomic DNA was extracted from rumen contents using the repeat bead-beating plus column method [57]. The quality and quantity of DNA were evaluated using a NanoDrop 2000 spectrophotometer (NanoDrop Technologies, Wilmington, DE, USA).

### Metagenome sequencing and data processing

Metagenome library construction was performed using TrueSeq DNA PCR-Free Library Prep Kits (Illumina, San Diego, CA, USA). Metagenome library sequencing was performed on an Illumina HiSeq 3000 platform (150 bp paired-end sequencing) at Majorbio Bioinformatics Technology Co., Ltd. (Shanghai, China).

The quality control of each dataset was performed using Sickle (version 1.33, https://github.com/najoshi/sickle). The 3′-ends of reads and 5′-ends of reads were trimmed, and the low-quality bases (quality score < 20), short reads (< 50 bp), and “N” records were removed. After quality control, the reads were aligned to the bovine genome (bosTau8 3.7, DOI: 10.18129/B9.bioc.BSgenome.Btaurus.UCSC.bosTau8) using BWA v0.7.1 (http://bio-bwa.sourceforge.net) to filter out host DNA [58]. The filtered reads were then de novo assembled using Megahit v1.1.2 (http://www.l3-bioinfo.com/products/megahit.html) [59]. The assembled contigs were annotated to open reading frames (ORFs) using MetaGene v0.3.38(http://metagene.cb.k.u-tokyo.ac.jp/) [60]. Assembled contigs were pooled, and non-redundancies were constructed using CD-HIT (95% identity, 90% coverage) (http://www.bioinformatics.org/cd-hit/) [61]. Original sequences were mapped to predicted genes (non-redundancies) to estimate their abundances using SOAPaligner v2.21 (http://soap.genomics.org.cn/) [62].

### ARG identification and resistome analysis

Contigs were annotated using DIAMOND v0.8.35 (http://ab.inf.uni-tuebingen.de/software/diamond) against the CARD Database v3.0.7 (https://card.mcmaster.ca) with an E value of 1e-5 and 90% coverage to identify the ARGs [63]. The CARD database provides a list of the antimicrobial resistance mechanisms, including a majority of 7 categories [63], and such information was utilized to identify the mechanisms of specific ARGs in our dataset. The classification of ARG classes and resistance mechanisms were utilized by Krona (https://github.com/marbl/Krona/wiki). The read counts within each sample were normalized into CPM for downstream analysis. The alpha diversity indices were calculated using the normalized read counts of ARGs. Beta diversity (principal component analysis [PCA]) using Bray-Curtis dissimilarity and RDA were performed based on the normalized read counts. Cows were clustered based on the rumen ARGs using the Jensen-Shannon distance and partitioning ARGund medoid (PAM) clustering [47]. The optimal number of clusters was estimated based on the Calinski-Harabasz (CH) index, and the clustering was represented using the principal coordinate analysis (PCoA) plot.

Taxonomic assessment of rumen microbiota was performed using DIAMOND 0.8.35 against the RefSeq database (http://www.ncbi.nlm.nih.gov/RefSeq/) using the contigs that harbour ARGs. Taxonomic profiles were generated at the phylum, family, genus, and species levels, and the relative abundances were calculated.

### Statistical analysis

All statistical analyses were performed in R (https://www.r-project.org). The DMI in the two groups in study 1 and milking traits in the two groups in study 2 were compared using the t-test. The PERMANOVA based on the abundances of the ARGs was performed with 1000 permutations to test the difference in rumen ARGs in cows with different phenotypes. The ARGs existing in at least 50% of cows within each group were used for downstream comparison analysis. The ARGs in different DMI groups were compared using the Wilcoxon rank-sum test, with a false discovery rate (FDR) < 0.05 considered significantly different. The ARGs in cows with different milk protein yields were compared using the Wilcoxon rank-sum test and linear discriminant analysis effect size (LEfSe), and significant differences were examined by linear discriminant analysis (LDA) score > 2 and *P* value < 0.05. The correlation analysis was performed using Spearman’s rank correlation, and a *P* value of Spearman’s coefficient < 0.05 was considered significant.

## Supplementary Information

**Additional file 1 : Table S1.** Summary of sequence data generated from rumen samples of 49 dairy cows.

**Additional file 2 : Table S2.** Sub-terms of ARGs linked to the mechanism of antimicrobial efflux detected in the current study.

**Additional file 3 : Table S3.** Comparison analysis of resistance genes in the rumen of dairy cows with different feed intake.

**Additional file 4 : Table S4**. Distributions of ARGs annotated to bacterial taxa in the rumen of cows with different milk protein yield.

**Additional file 5 : Table S5**. Comparison analysis of resistance genes in the rumen of dairy cows with different milk protein yield.

**Additional file 6 : Table S6**. The predominant resistance genes of the four resistotypes.

**Additional file 7 : Figure S1.** Microbial profiles of 49 dairy cows. Compositional profile of microbial domains (A). Compositional profile of bacterial phyla (B).

**Additional file 8 : Figure S2**. Comparison of bacteriome between the two DMI groups. Principal component analysis (A) and clustering of samples (B) based on relative abundances of bacterial species. Relative abundances of top 20 bacterial species (C).

**Additional file 9 : Figure S3.** Ruminal resistome profiles of cows with different milking traits. Principal component analysis for ARGs calculated based on counts per million. The colours in the PCA plots show cows with different milking performances, including milk protein content (A), milk protein yield (B), milk fat content (C), and lactose content (D).

**Additional file 10 : Figure S4**. Comparison of bacteriome between HH and LL groups. Principal component analysis (A) and clustering of samples (B) based on relative abundances of bacterial species. Relative abundances of top 20 bacterial species (C).

**Additional file 11 : Figure S5**. Functional comparison of rumen microbiome between HH and LL groups. The HH/LL fold change shows differences in level-3 microbial pathways between HH and LL cows, including amino acid metabolism (A), carbohydrate metabolism (B), metabolism of cofactors and vitamins (C) and energy metabolism (D).

**Additional file 12 : Figure S6**. Stratification of the ruminal resistome composition in study 1. The principal coordinate analysis of the ruminal resistome showed resistance types (resistotypes) among the 33 dairy cows in study 1. Animals were divided into two groups based on dry matter intake (DMI).

## Data Availability

The rumen metagenome sequences were deposited into the NCBI Sequence Read Archive (SRA) under the accession numbers PRJNA526070 (https://www.ncbi.nlm.nih.gov/bioproject/PRJNA526070) and PRJNA597489 (https://www.ncbi.nlm.nih.gov/bioproject/PRJNA597489).
