# Supplementary figures and images for: Ruminal resistome of dairy cattle is individualized and the resistotypes are associated with milking traits
Source: Anim Microbiome. 2021 Feb 10;3:18. doi: 10.1186/s42523-021-00081-9 (PMC7877042; doi:10.1186/s42523-021-00081-9)

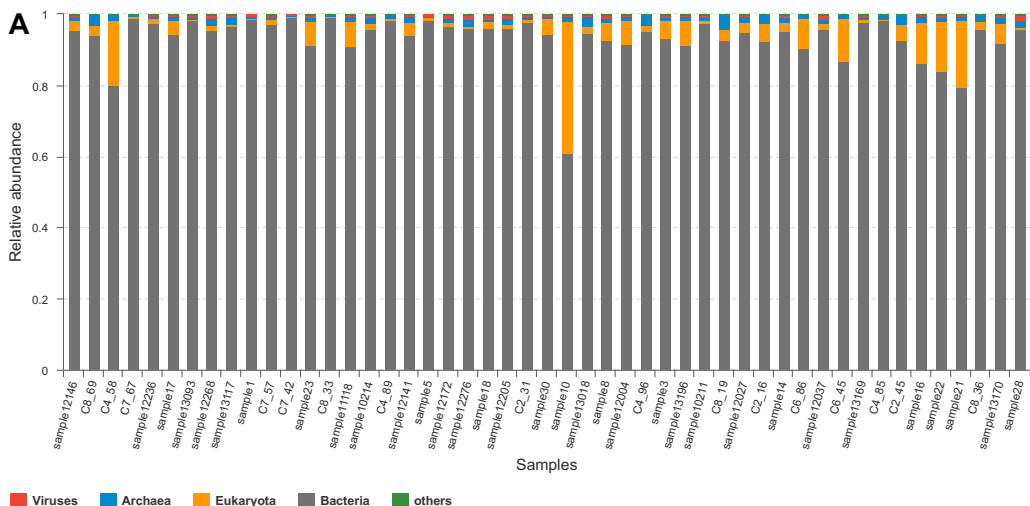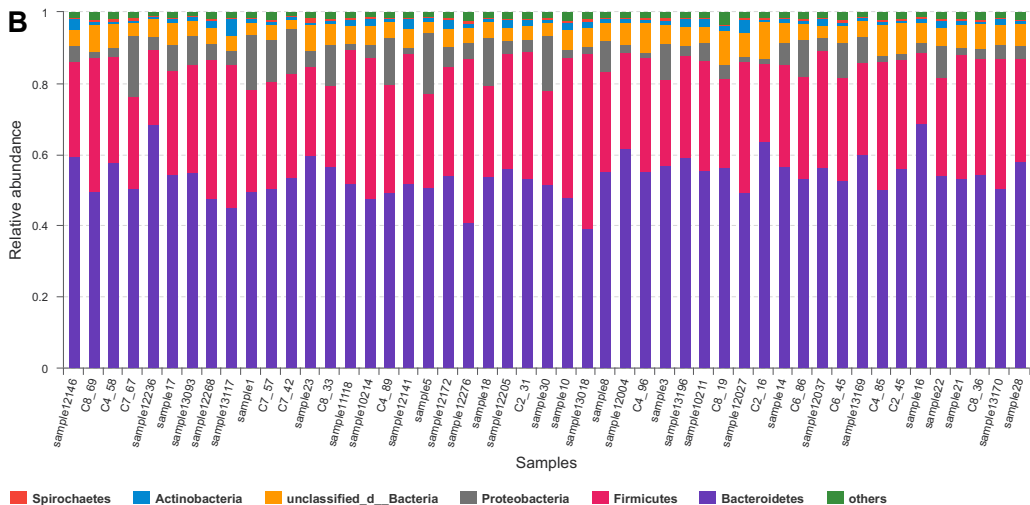

Supplement: Supplementary file 7 — Additional file 7 : Figure S1. Microbial profiles of 49 dairy cows. Compositional profile of microbial domains (A). Compositional profile of bacterial phyla (B). [file 42523_2021_81_MOESM7_ESM.pdf]

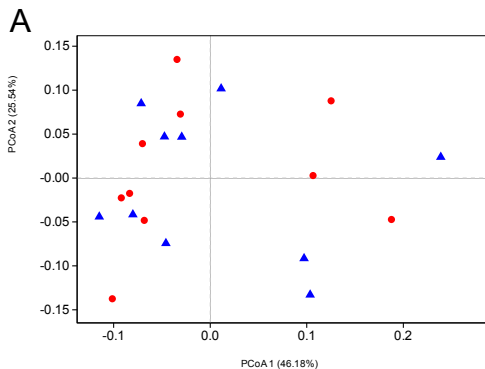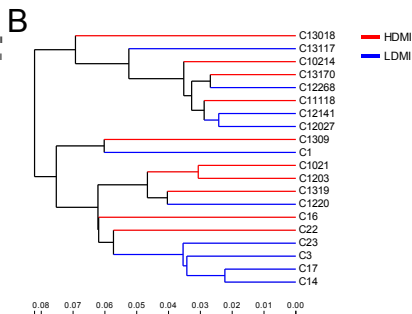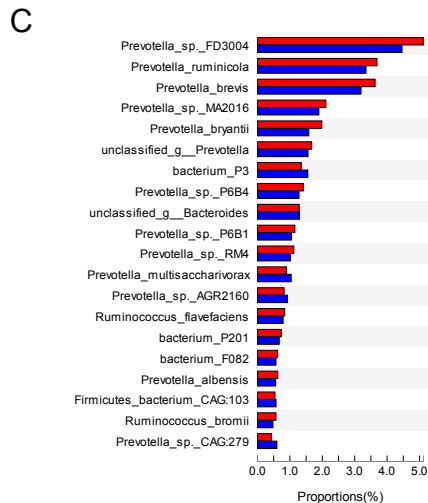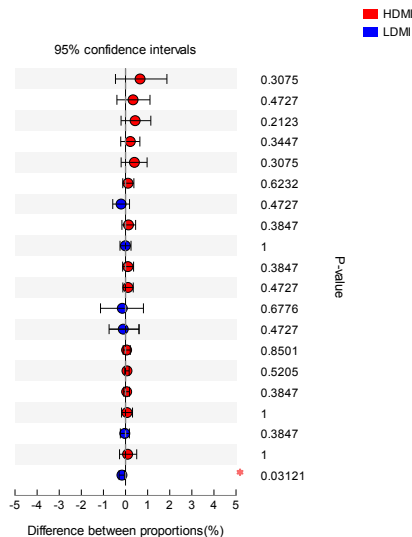

Supplement: Supplementary file 8 — Additional file 8 : Figure S2. Comparison of bacteriome between the two DMI groups. Principal component analysis (A) and clustering of samples (B) based on relative abundances of bacterial species. Relative abundances of top 20 bacterial species (C). [file 42523_2021_81_MOESM8_ESM.pdf]

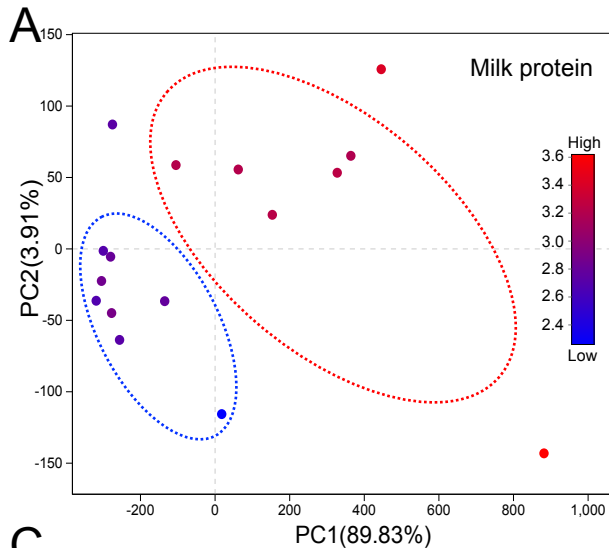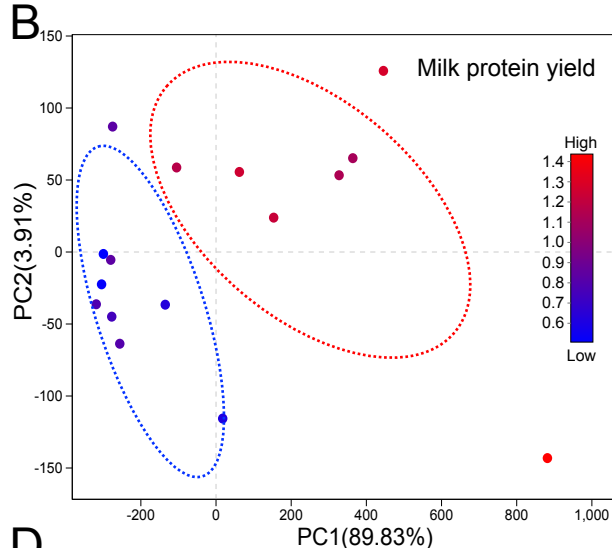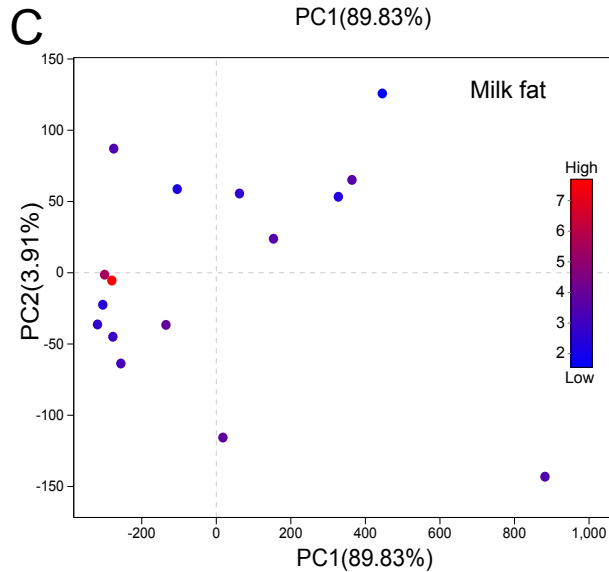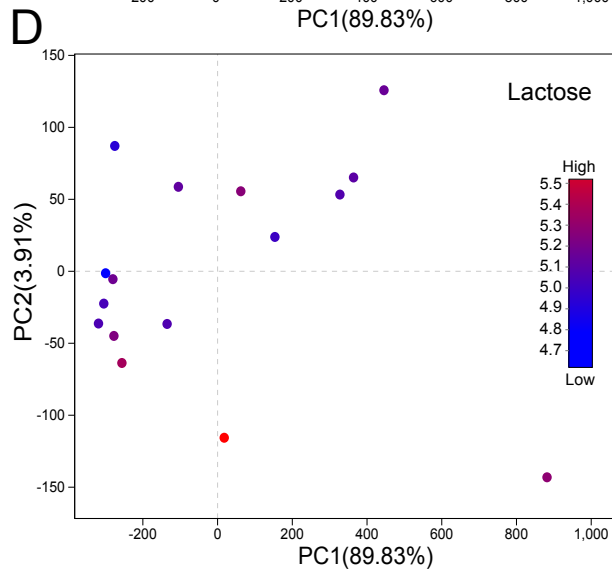

Supplement: Supplementary file 9 — Additional file 9 : Figure S3. Ruminal resistome profiles of cows with different milking traits. Principal component analysis for ARGs calculated based on counts per million. The colours in the PCA plots show cows with different milking performances, including milk protein content (A), milk protein yield (B), milk fat content (C), and lactose content (D). [file 42523_2021_81_MOESM9_ESM.pdf]

A

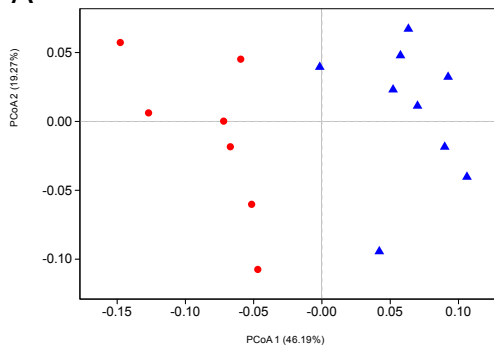

B

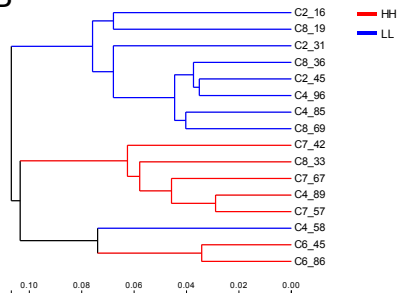

C

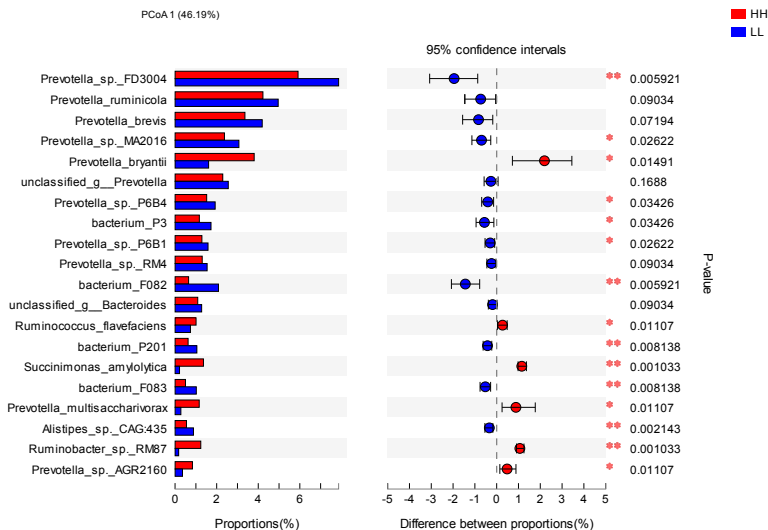

Supplement: Supplementary file 10 — Additional file 10 : Figure S4. Comparison of bacteriome between HH and LL groups. Principal component analysis (A) and clustering of samples (B) based on relative abundances of bacterial species. Relative abundances of top 20 bacterial species (C). [file 42523_2021_81_MOESM10_ESM.pdf]

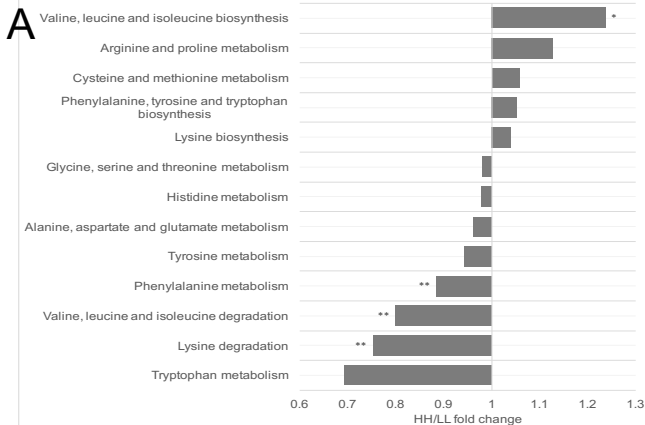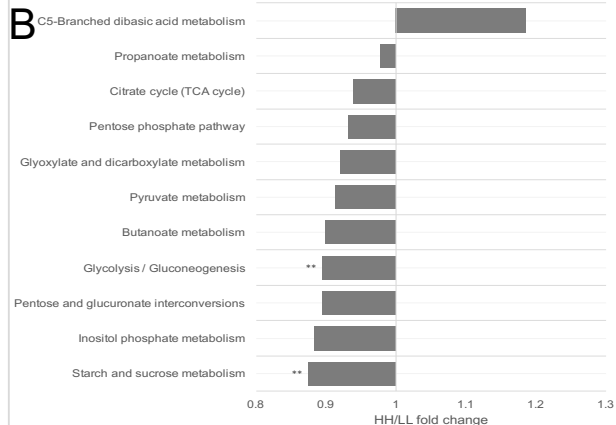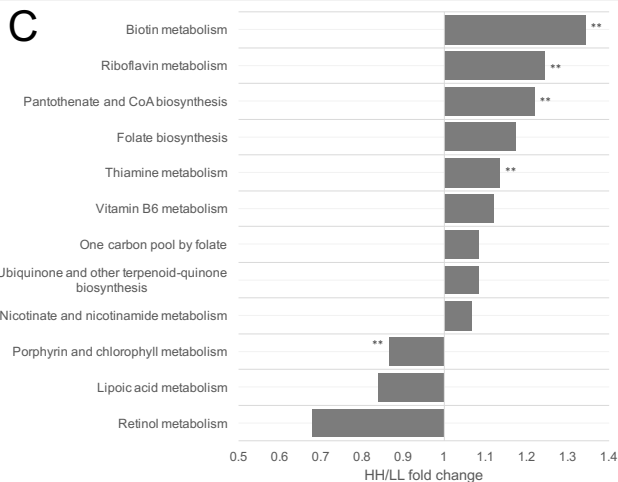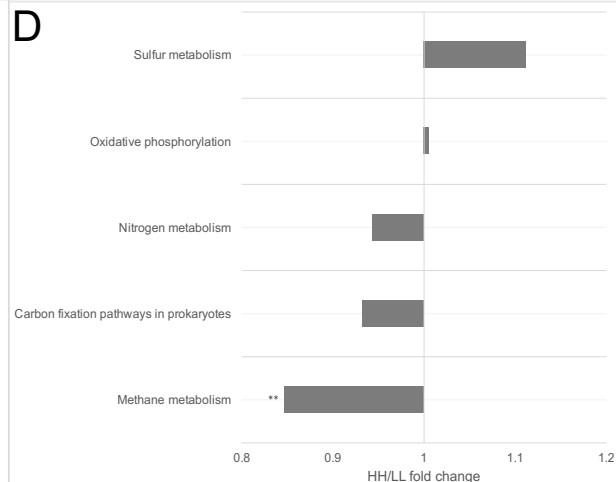

Supplement: Supplementary file 11 — Additional file 11 : Figure S5. Functional comparison of rumen microbiome between HH and LL groups. The HH/LL fold change shows differences in level-3 microbial pathways between HH and LL cows, including amino acid metabolism (A), carbohydrate metabolism (B), metabolism of cofactors and vitamins (C) and energy metabolism (D). [file 42523_2021_81_MOESM11_ESM.pdf]

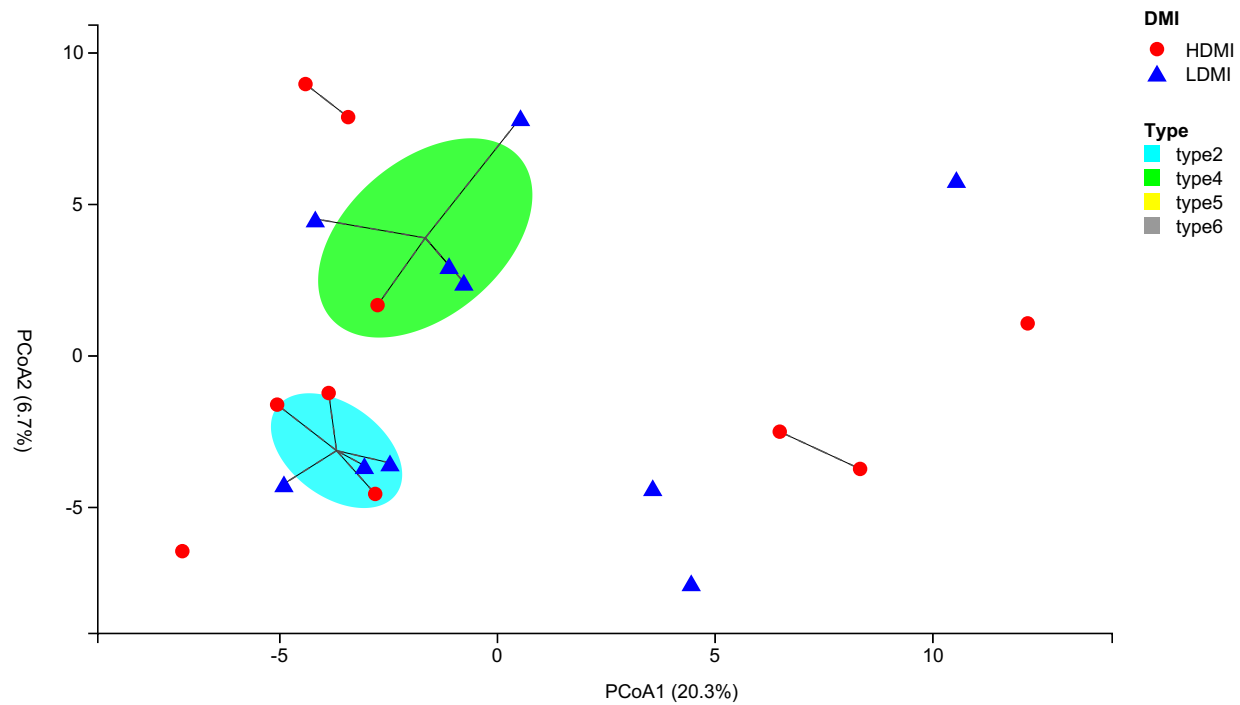

Supplement: Supplementary file 12 — Additional file 12 : Figure S6. Stratification of the ruminal resistome composition in study 1. The principal coordinate analysis of the ruminal resistome showed resistance types (resistotypes) among the 33 dairy cows in study 1. Animals were divided into two groups based on dry matter intake (DMI). [file 42523_2021_81_MOESM12_ESM.pdf]
